# Supplementary material for: Platonic representation of foundation machine learning interatomic potentials
Source: Nat Mach Intell. 2026 May 7;8(5):830–40. doi: 10.1038/s42256-026-01235-7 (PMC13201161; doi:10.1038/s42256-026-01235-7)
Supplement: Supplementary file 1 — Supplementary Figs. 1–7, Discussion and Tables 1–7. [file 42256_2026_1235_MOESM1_ESM.pdf]

nature machine intelligence

Supplementary information https://doi.org/10.1038/s42256-026-01235-7

Platonic representation of foundation machine learning interatomic potentials

In the format provided by the authors and unedited

Table of Contents

1. Non-linear projection of embeddings (Fig. S1)
2. Justification of anchor selection strategy (Table S1)
3. Robustness of anchor-based transformation (Fig. S2)
4. Evaluate the converged form of unified representations (Table S2)
5. Platonic representation holds for extended models (Fig. S3)
6. Supplement metrics (Fig. S4, S5)
7. Unified embedding arithmetic (Tables S3, S4)
8. Re-discovered equivariant atoms by Platonic representation (Tables S5, S6, S7)
9. Dummy MACE model
10. Technical report for the extraction of embeddings (Figs. S6, S7)
11. Manifold Distance

1. Non-linear projection of embeddings

We have down-selected 5000 embeddings randomly from the full phenomenological embedding space and adopted the UMAP visualization. No clear Platonic nature being captured by the non-linear projection of embeddings.

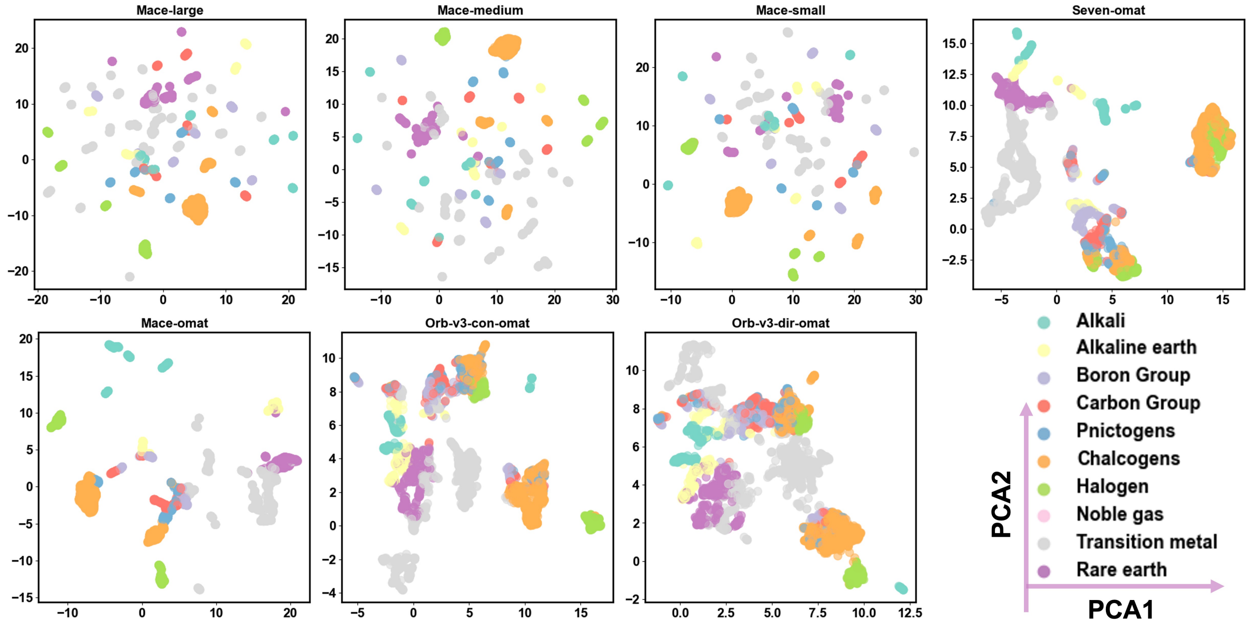

Fig. S1: UMAP visualization of the seven chosen MLIPs, with random_state=42, n_neighbors=30.

2. Justification of Anchor selection strategy

We compared the diversity and clustering of anchor sets obtained both from random sampling and the DIRECT Sampling strategy.

Table S1: Silhouette scores and pairwise distances (in parentheses) for anchor sets selected using different strategies. For random sampling, the values correspond to runs with random seeds 0, 42, and 12345. For DIRECT Sampling, the values correspond to threshold_init settings of 0.1, 0.2, and 0.3. When evaluating clusterability using KMeans, the number of target clusters is set to 10, matching the number of labeled elemental groups. A lower Silhouette score indicates a less clusterable anchor set, whereas a larger pairwise distance indicates a more diverse anchor set.

Size of Anchor set	Random Sampling	DIRECT Sampling
100	0.09 (0.4), 0.14(0.1), 0.14(0.45)	0.14(0.62), 0.14(0.47), **0.06(1.73)**
200	0.17(0.4), 0.17(0.1), 0.16(0.43)	**0.08(1.47)**, 0.06(0.84), 0.09(0.79)
400	0.12(0.1), 0.14 (0.1), 0.13(0)	0.07(0.59), **0.09(0.74)**, 0.10(0.31)

3. Robustness of anchor-based transformation

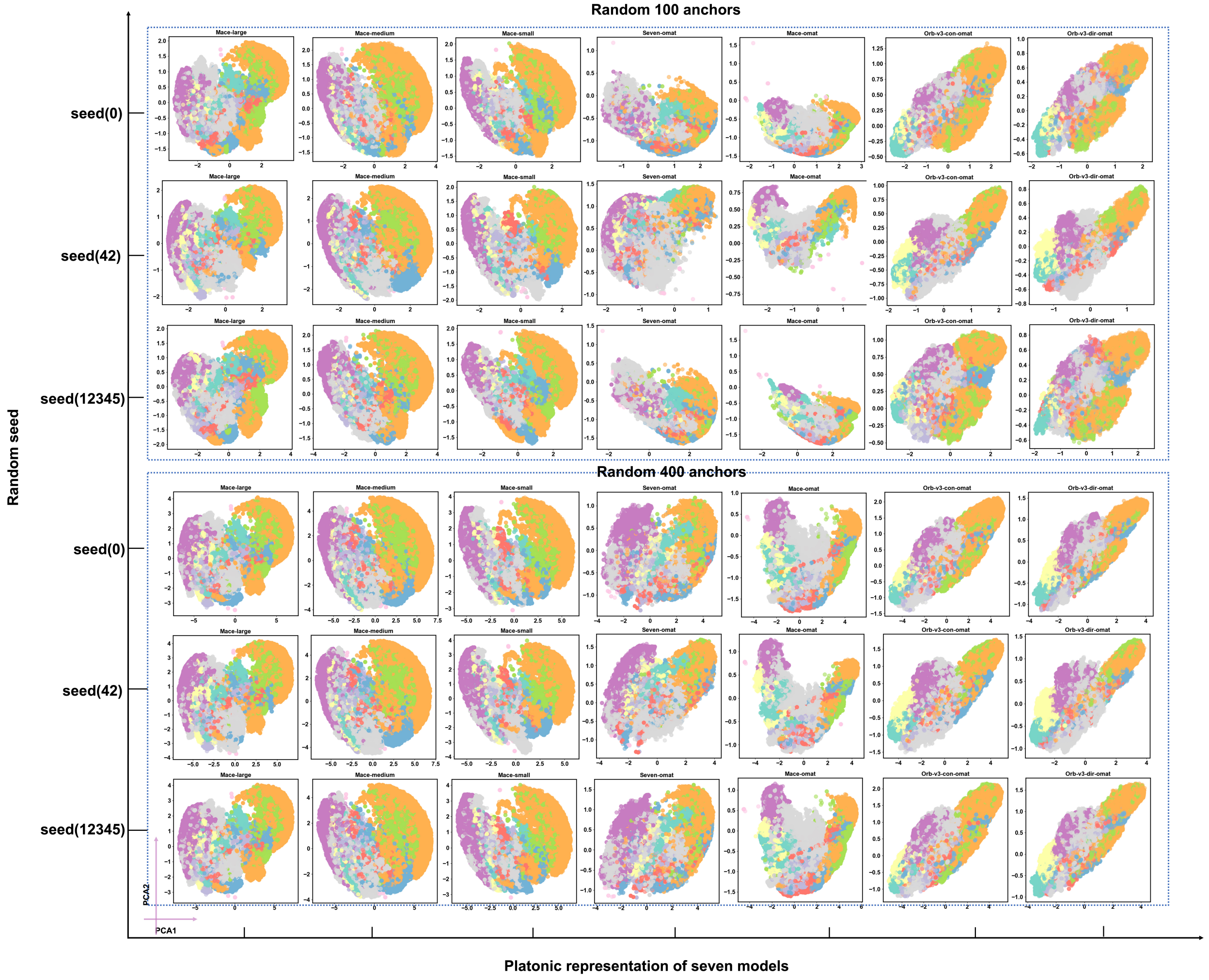

Fig. S2: The robustness of anchor-based transformation tested against different random seeds.

4. Evaluate the converged form of unified representations

To quantify the clustering score of converged representations after transformation for both anchor selection strategies, the Silhouette scores (S) and mean pairwise distances (D) were computed, where smaller mean pairwise distances (D) means more compact clustering after transformation.

Results in Table S2 show that, DIRECT sampling generated more compact clustering with smaller D in contrast to the values from random sampling. Both converged representations show similar Silhouette scores (S) for each model.

Table S2: Silhouette scores (S) and mean pairwise distances (D) for the converged transformed embeddings using random and DIRECT sampling. Using random seed 42 to down selected 5000 embeddings.

	large	medium	small	7omat	Mace-omat	Orb-v3-con-omat	Orb-v3-dir-omat
S(Random)	0.30	0.28	0.29	0.22	0.28	0.16	0.12
S(DIRECT)	0.28	0.26	0.27	0.23	0.26	0.15	0.11
D(Random)	3.06	3.11	2.71	2.18	2.20	2.28	1.80
D(DIRECT)	**2.60**	**2.67**	**2.34**	**2.10**	**2.08**	**2.00**	**1.58**

5. Platonic representation holds for extended models

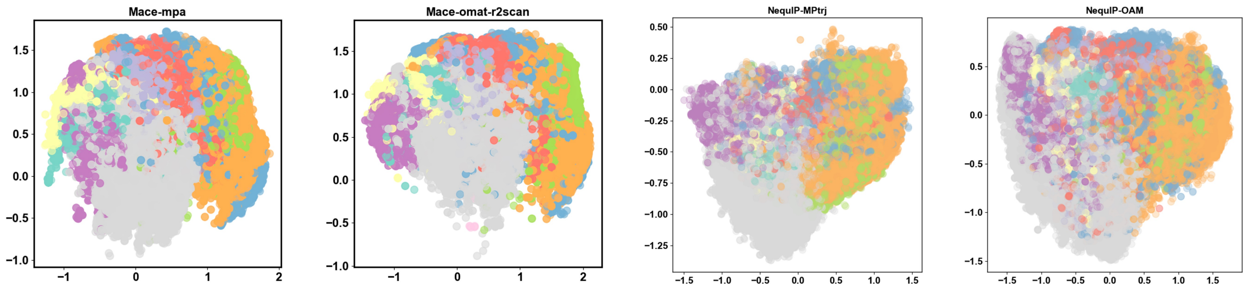

Fig. S3: The unified representation for foundational MACE-MPA-0, MACE-omat-r2scan, NequIP-MPtrj and NequIP-OAM models.

6. Supplement metrics

We have used the mKNN to measure the local arrangement of embeddings to measure the neighborhood overlap for each sample; for the CKA measurement, it is global and measures the overall pairwise relationship alignment, therefore, this measure requires dimensionality match; and it is used to compare with the Procrustes score, which is also global and measure the best rotation/alignment distance.

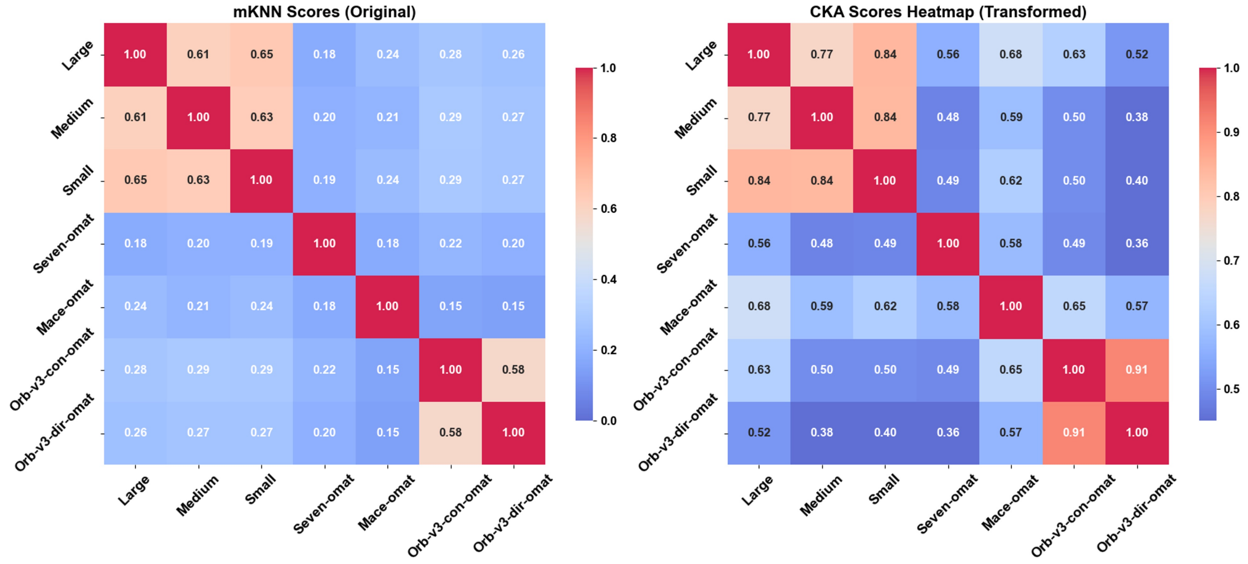

Fig. S4: (Left) mKNN performed on the original embedding space. (Right) The unified representation enables the CKA measurement, which shows consistent results as the $\text{Score}_{\text{Procrustes}}$.

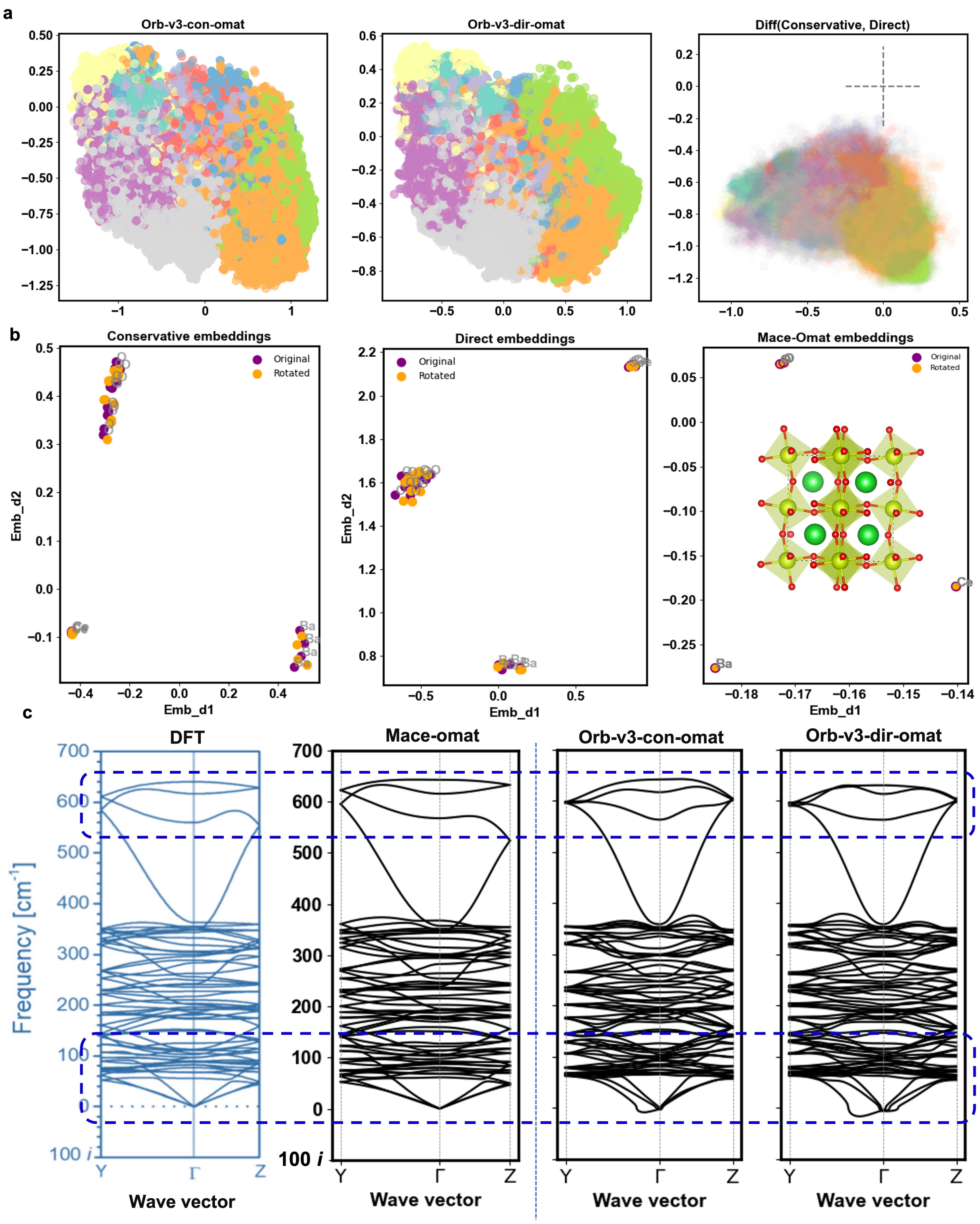

Fig. S5: **Detecting symmetry breaking.** (a) PCA projection of the difference vector between Orb-v3 conservative and non-conservative embeddings. (b) Rotational sensitivity test on $BaCeO_3$: Equivariant models preserve embeddings under rotation; Orb-v3 models do not. (c) These representational errors propagate to physical predictions, resulting in incorrect phonon dispersions and imaginary acoustic modes close to Γ.

7. Unified embedding arithmetic

$\mathbf{Emb_{Mater}}$: Extended set of materials and the ls of their $\mathrm{Emb_{Mater}}$, where scaling factor1 is the l from the Seven-omat relative to that from the Mace-large model and the scaling factor2 is the l from the Mace-omat relative to that from the Mace-large model.

Table S3: Scaling factor between Mace-omat, Seven-omat models relative to the Mace-large models.

	Seven-omat	Mace-omat	large	scaling factor1	scaling factor2
$Na_3MnCoNiO_6$	4.50	5.44	1.14	2.94	3.56
$Nd(Al_2Cu)_4$	6.17	6.32	1.66	2.88	2.96
$LiMnIr_2$	5.17	6.35	1.50	3.00	3.68
LiCSN	5.45	6.36	1.49	3.24	3.78
La_2EuS_4	5.71	6.31	1.89	2.71	2.99
$Yb_3Ga_9Pt_2$	6.08	6.31	1.93	2.79	2.90
$NaLiCO_3$	5.34	5.95	1.56	4.06	4.52
Na_2BiO_3	5.09	5.68	1.59	3.23	3.61

$\mathbf{Emb_{React}}$: Extended set of hypothetical reactions to check the cross-model consistency of $\mathrm{Emb_{React}}$ and the zero-shot stitching compatibility between all the other models with the Mace-large model.

Table S4: R1: $BaO + ZrO_2 \rightarrow BaZrO_3$; R2: $2CsF + InF_3 + AgF \rightarrow CsInAgF_6 + Cs$; R3: $CsCl + SnCl_2 \rightarrow CsSnCl_3$, where for each reaction we list the reaction embeddings from intra-model and stitched model with Mace-small, with respect to (l, c-sim).

MLIPs	R1	Stitch-R1	R2	Stitch-R2	R3	Stitch-R3
Mace-large	1.31, 1	1.78, 1	3.57, 1	3.34, 1	1.86, 1	1.64, 1
Mace-medium	1.11, 0.82	2.01, 0.91	3.08, 0.85	4.51, 0.95	1.48, 0.86	2.15, 0.95
Mace-small	1.30, 0.87	1.30, 0.84	3.16, 0.92	3.16, 0.93	1.74, 0.97	1.74, 0.96
Seven-omat	5.43, 0.76	3.44, 0.34	16.66, 0.73	3.85, 0.51	5.72, 0.82	3.06, 0.17
Mace-omat	5.71, 0.75	4.16, 0.39	14.73, 0.72	4.60, 0.35	5.45, 0.86	3.24, 0.32
Orb-v3-con-omat	1.17, 0.51	2.72, 0.84	2.80, 0.58	5.97, 0.91	0.91, 0.33	3.37, 0.97
Orb-v3-dir-omat	1.48, 0.67	2.27, 0.63	4.14, 0.74	4.81, 0.91	1.60, 0.86	3.56, 0.97

8. Re-discovered equivariant atoms by Platonic representation

We have collected all the embeddings pairs that have distances below 1E-8 as the ground truth of equivariant pairs.

Table S5: Count of equivariant atoms based on brute force distance calculation, denoted as $\frac{\text{Count}_{\text{Original}}.}{\text{Count}_{\text{Platonic}}}$. Accuracy: 1E-8

space group	large	medium	small	Mace-omat	Seven	NequIP	orb-con	orb-dir
221	$\frac{2674}{2674}$	$\frac{2674}{2674}$	$\frac{2674}{2674}$	$\frac{2674}{2674}$	$\frac{3}{3}$	$\frac{2132}{2}$	$\frac{0}{0}$	$\frac{0}{0}$
225	$\frac{22920}{22922}$	$\frac{22945}{22925}$	$\frac{22920}{22928}$	$\frac{22942}{22946}$	$\frac{2}{2}$	$\frac{15720}{10}$	$\frac{0}{0}$	$\frac{0}{0}$
139	$\frac{4878}{4896}$	$\frac{4896}{4896}$	$\frac{4877}{4896}$	$\frac{4881}{4881}$	$\frac{4}{4}$	$\frac{3625}{0}$	$\frac{0}{0}$	$\frac{0}{0}$
194	$\frac{9248}{9252}$	$\frac{9248}{9252}$	$\frac{9247}{9252}$	$\frac{9248}{9252}$	$\frac{189}{189}$	$\frac{9410}{2}$	$\frac{0}{0}$	$\frac{0}{0}$

Table S6: Count of equivariant atoms based on brute force distance calculation, denoted as $\frac{\text{Count}_{\text{Original}}.}{\text{Count}_{\text{Platonic}}}$. Accuracy: 1E-6

space group	large	medium	small	Mace-omat	Seven	NequIP	orb-con	orb-dir
221	$\frac{2677}{2678}$	$\frac{2678}{2677}$	$\frac{2677}{2678}$	$\frac{2678}{2678}$	$\frac{2663}{2673}$	$\frac{2733}{2629}$	$\frac{0}{0}$	$\frac{0}{0}$
225	$\frac{23308}{23325}$	$\frac{23327}{23318}$	$\frac{23288}{23308}$	$\frac{23334}{23350}$	$\frac{22975}{23285}$	$\frac{27162}{23379}$	$\frac{0}{0}$	$\frac{0}{0}$
139	$\frac{4993}{5011}$	$\frac{5027}{5013}$	$\frac{4990}{5004}$	$\frac{5025}{5076}$	$\frac{4867}{4988}$	$\frac{5614}{5193}$	$\frac{0}{0}$	$\frac{0}{0}$
194	$\frac{9831}{10670}$	$\frac{10749}{10770}$	$\frac{9668}{10864}$	$\frac{9820}{11151}$	$\frac{9134}{10191}$	$\frac{15304}{13617}$	$\frac{0}{0}$	$\frac{0}{0}$

Table S7: Count of re-discovered equivariant atoms by Platonic representation, denoted as $\frac{\text{Count}_{\text{Original}}}{\text{Count}_{\text{Platonic}}}$ with the two-NN algorithm.

space group	large	medium	small	Mace-omat	Seven	NequIP	orb-con	orb-dir
221	$\frac{1247}{1389}$	$\frac{1283}{1316}$	$\frac{1228}{1354}$	$\frac{1289}{1364}$	$\frac{0}{286}$	$\frac{14}{628}$	$\frac{0}{0}$	$\frac{0}{0}$
225	$\frac{9098}{9279}$	$\frac{9065}{9091}$	$\frac{8941}{9160}$	$\frac{9184}{9031}$	$\frac{4}{2470}$	$\frac{59}{3433}$	$\frac{0}{0}$	$\frac{0}{0}$
139	$\frac{3356}{3429}$	$\frac{3293}{3343}$	$\frac{3301}{3500}$	$\frac{3202}{3428}$	$\frac{0}{492}$	$\frac{4}{810}$	$\frac{0}{0}$	$\frac{0}{0}$
194	$\frac{5276}{5212}$	$\frac{5190}{5393}$	$\frac{5288}{5367}$	$\frac{20}{499}$	$\frac{13}{2363}$	$\frac{14}{628}$	$\frac{0}{0}$	$\frac{0}{0}$

9. Dummy MACE model

Listing 1: Randomizing MACE-small foundation model

```
import torch
from mace.calculators import mace_mp
# Get the foundation model with return_raw_model=True
model = mace_mp(model="small", device="cuda", return_raw_model=True)
# Randomize all weights
for param in model.parameters():
    if param.requires_grad:
        torch.nn.init.normal_(param, mean=0.0, std=0.02)
# Save the randomized model
torch.save(model, "dummy_mace_mp_small_randomized.model")
print(f"Randomized model with {sum(p.numel() for p in model.parameters())}
    ↪ parameters")
```

10. Technical report for the extraction of embeddings

Layer for embedding extraction

For MACE models, we call get_descriptors() with num_layers = 1 and invariants_only = True to get the rotationally invariant (L=0) embeddings from the first interaction layer of the MACE model. MACE's built-in get_descriptors() API computes node-level ACE (Atomic Cluster Expansion) descriptors that aggregate message-passing information from num_layers interaction layers. Using the official API is the most reliable way to get physically meaningful, rotationally-invariant descriptors. The num_layers = 1 captures local environment; increasing it expands the receptive field.

For SevenNet models, extracted from the layer immediately before reduce_input_to_hidden or readout_FCN — i.e., the final equivariant convolution/gate layer, identified dynamically at

runtime via forward hooks. This is the last hidden representation before the energy readout head. It contains the richest structural encoding after all message-passing rounds, but hasn't yet been projected down to a scalar energy. The code searches for reduce_input_to_hidden or readout_FCN and takes the layer just before it; if neither is found, it falls back to the last equivariant_gate or convolution layer. The hook captures node_feature (or .x) from that layer's output. For SevenNet, the last interaction block is architecturally constrained to output only l=0 (scalar) features (lmax_node=0), making the hooked representation rotationally invariant by construction

For Orb models, extracted from the final GNN output, accessed via result[“node_features”] from the _gns_model forward pass. Orb's architecture exposes “node_features” as a named output key after the full GNN stack. This is the most semantically complete per-atom representation — after all message-passing — before the force/energy decoder. It's the cleanest and most direct API-level access Orb provides.

For NequIP models, extracted from different convolution layer, captured by inserting SaveForOutput hook modules into all layers matching “layer” +“convnet” in the name.

Rationale of layer choice

Our rule for selection is the invariant (l=0) component of embeddings before energy readout. In MACE, num_layers in get_descriptors() controls the radial reach of the descriptor — how many interaction blocks of neighborhood information to aggregate. It's more analogous to a cutoff depth in a graph expansion than to “which hidden layer to tap”. Therefore, we didn't observe obvious change of representations from layer 1 to layer 12 (Fig. S6). On the other hand, SevenNet/Orb/NequIP's layer choice is about network depth — literally which intermediate feature tensor to extract from the forward pass, with deeper layers being more processed/abstract. For SevenNet and Orb, we follow the common practice in the MLIP community of extracting from the final node feature layer immediately before the energy readout head, which captures the richest structural encoding after full message passing while avoiding distortion by the task-specific decoder.

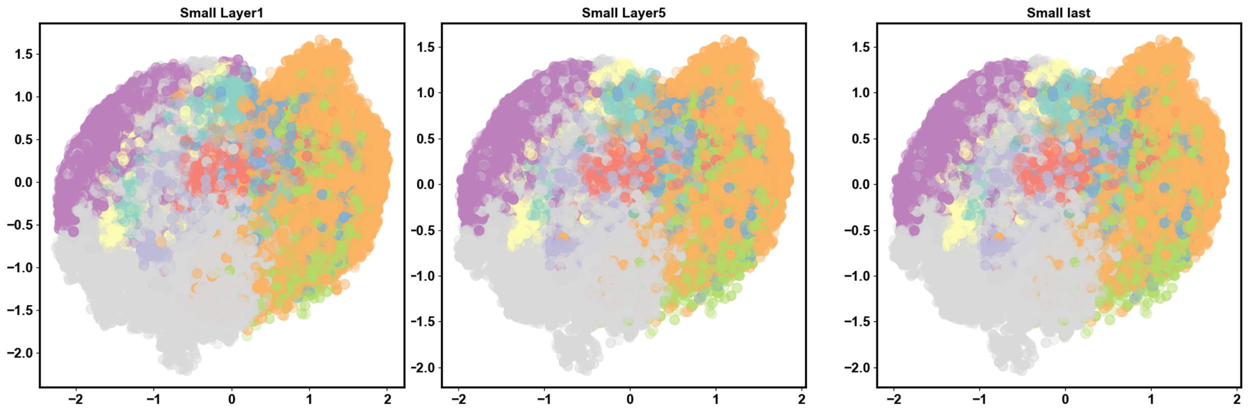

Fig. S6: **MACE representations extracted by setting num layers as 1, 5 and 12 (last layer).**

Unlike SevenNet and Orb, the node representations in NequIP change substantially with network depth (Fig. S7). Only the embeddings from the first three convolutional layers bear resemblance to those produced by the other models; deeper layers become increasingly task-specific. We therefore extract embeddings (l=0, invariant part) after the 2nd convolutional layer for NequIP, balancing sufficient message-passing context with representational consistency across models.

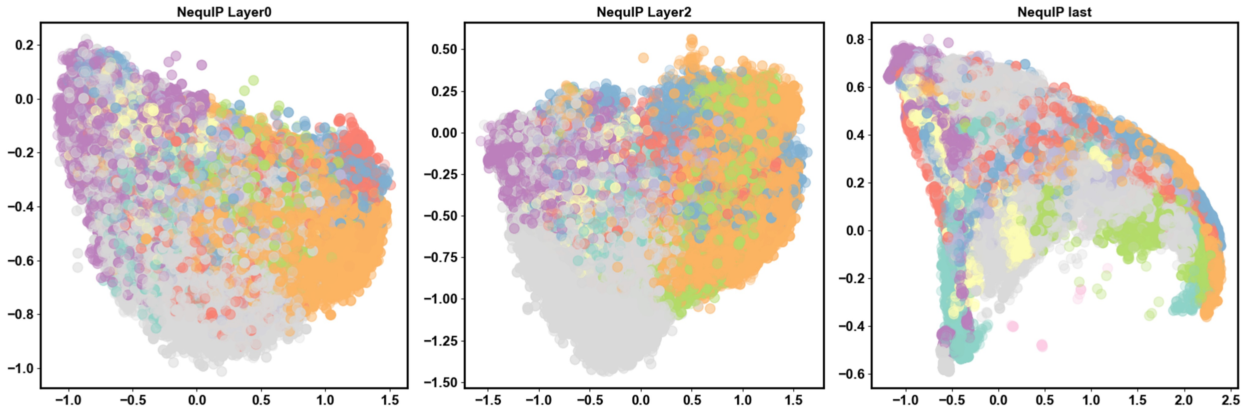

Fig. S7: **NequIP representations extracted from convolutional layers 0, 2 and the last layer before energy readout.**

Equivariant vs Invariant representations

MACE, SevenNet are equivariant architectures. We extracted the invariant part related with energy predictions. MACE's get_descriptors(invariants_only=True) returns explicitly

invariant descriptors. For SevenNet, the last interaction block is architecturally constrained to output only l=0 (scalar) features (lmax_node=0), making the hooked representation rotationally invariant by construction. We extract from this final node feature layer, immediately before the energy readout MLP. For Orb models, the final node feature representations are extracted directly from the GNN output layer as returned by the model, without additional processing.

On fair comparison The layer-depth choice can potentially introduce a source of variation, even though our choice of layer tries to mitigate this influence for the models discussed above. However, in the future, we hope a more information theory-based way of selection can be developed.

From atom-level to material-level embeddings

The embeddings we extract are per-atom (i.e., context-dependent: the same element in different chemical environments yields different embeddings), not per-element-type. When we visualise “element-level” embeddings (Fig. 4e-f), we are showing the distribution of mean-pooled element-level embeddings across all the atomic environments that an atom has presented. The material-level vector defined in Eq. 3 is the centroid of all per-atom embeddings in a structure—a mean-pooled aggregate—and this is also now stated more clearly.

11. Manifold Distance

Each generated structure is assigned a scalar descriptor by mean-pooling the per-atom embeddings extracted from the MLIP model (described above), yielding one vector per structure. The reference manifold is defined by the embeddings of the training dataset — the region of chemical space occupied by known stable materials, MP20 here. Five geometric and statistical metrics are computed for each generated structure with respect to this manifold. Code provided at `https://github.com/WMD-group/PlatonicRep`.

1. Manifold distance. A k-nearest-neighbour (kNN) graph (k = 10) is fitted to the reference embeddings using the Euclidean metric. For each generated structure, the mean distance to its k nearest reference neighbours is computed and z-score normalised against the reference self-distance distribution, giving a dimensionless manifold distance.

2. Depth score. The Euclidean distance from each generated structure to the centroid of the reference set, normalised by the maximum centroid distance observed within the reference, provides a measure of how central (low score) or peripheral (high score) a structure is relative to the core of the training manifold.

3. Boundary distance. For high-dimensional embedding spaces, a one-class Support Vector Machine (OneClassSVM, RBF kernel, $\nu = 0.1$) is fitted to the standardised reference embeddings. Its decision function, normalised by the intra-reference standard deviation, provides a signed distance to the learned manifold boundary (positive = inside, negative = outside). For low-dimensional spaces ($\leq$3 dimensions), a Delaunay triangulation is used instead.

4. Local density and density percentile. Local density at each generated structure is estimated as the reciprocal of the mean kNN distance. This value is ranked against the empirical reference density distribution to produce a density percentile, indicating how populated the local neighbourhood is relative to the typical reference density.

5. Local Outlier Factor (LOF). A novelty-mode LOF model (k = 10, contamination = 0.1) is fitted to the reference embeddings. LOF scores close to -1 indicate local densities consistent with the reference; strongly negative values (below the threshold of -2) indicate genuine outliers.

Structures are then partitioned into four categories. i) Dense interior: inside the boundary, density percentile $\geq$ 20th percentile, LOF $\geq$ -2 (well-supported candidates including near-exact matches to reference). ii) Sparse interior: inside the boundary, density percentile $<$ 20th percentile, LOF $\geq$ -2 (novel but locally consistent). iii) Near exterior: outside the boundary, LOF $\geq$ -2 (exploration candidate with no nearby reference). iv) Far exterior:

LOF score < -2, regardless of position or local density (locally inconsistent with the reference distribution).
