# Supplementary material for: Platonic representation of foundation machine learning interatomic potentials
Source: Nat Mach Intell. 2026 May 7;8(5):830–40. doi: 10.1038/s42256-026-01235-7 (PMC13201161; doi:10.1038/s42256-026-01235-7)
Supplement: Supplementary file 2 — Reporting Summary [file 42256_2026_1235_MOESM2_ESM.pdf]

nature portfolio

Corresponding author(s): Dr. Zhenzhu Li

Last updated by author(s): Apr 6, 2026

Reporting Summary

Nature Portfolio wishes to improve the reproducibility of the work that we publish. This form provides structure for consistency and transparency in reporting. For further information on Nature Portfolio policies, see our Editorial Policies and the Editorial Policy Checklist.

Statistics

For all statistical analyses, confirm that the following items are present in the figure legend, table legend, main text, or Methods section.

n/a	Confirmed	
☐	☒	The exact sample size (n) for each experimental group/condition, given as a discrete number and unit of measurement
☐	☒	A statement on whether measurements were taken from distinct samples or whether the same sample was measured repeatedly
☒	☐	The statistical test(s) used AND whether they are one- or two-sided<br>*Only common tests should be described solely by name; describe more complex techniques in the Methods section.*
☐	☒	A description of all covariates tested
☐	☒	A description of any assumptions or corrections, such as tests of normality and adjustment for multiple comparisons
☐	☒	A full description of the statistical parameters including central tendency (e.g. means) or other basic estimates (e.g. regression coefficient) AND variation (e.g. standard deviation) or associated estimates of uncertainty (e.g. confidence intervals)
☒	☐	For null hypothesis testing, the test statistic (e.g. F, t, r) with confidence intervals, effect sizes, degrees of freedom and P value noted<br>*Give P values as exact values whenever suitable.*
☒	☐	For Bayesian analysis, information on the choice of priors and Markov chain Monte Carlo settings
☒	☐	For hierarchical and complex designs, identification of the appropriate level for tests and full reporting of outcomes
☒	☐	Estimates of effect sizes (e.g. Cohen's d, Pearson's r), indicating how they were calculated

Our web collection on statistics for biologists contains articles on many of the points above.

Software and code

Policy information about availability of computer code

Data collection

1. Source: MP-20 dataset (publicly available, https://doi.org/10.6084/m9.figshare.25563693); 27,136 crystal structures from the Materials Project; Target set for atomic embedding extraction across all models.
2. Self-Generated Data: Atomic Embeddings (Main experimental data); Generated: 282,847 atomic embeddings per model;Models: 7 foundation MLIPs (MACE-large, MACE-medium, MACE-small, Seven-omat, MACE-omat, Orb-v3-con-omat, Orb-v3-dir-omat); Zenodo DOI: 10.5281/zenodo.17721681 (for reproducibility)
3. Anchor Sets: Self-generated via DIRECT sampling strategy
4. Control Data: self-constructed dummy model: MACE-small architecture with 3,847,696 randomized parameters (no training)
5. OMAT data, randomly selected 10000 rattled relax, 10000 at 300K, 10000 at 1000K
6. Data from generated models, 10000 each.

Data analysis

Machine learning foundation models for embedding extraction:
MACE-MP-0a: large, medium, small; MACE-Omat-0-medium model; SevenNet-Omat; orb_v3_conservative_inf_omat; orb_v3_direct_inf_omat
Anchor set selection: maml: v2025.4.1
Platonic projection: self-written code, provide in GitHub repo.
Embedding similarity metrics:
1. similarity-repository, v0.1.0
2. POT: 0.9.6
Plot: self-written code using matplotlib

For manuscripts utilizing custom algorithms or software that are central to the research but not yet described in published literature, software must be made available to editors and reviewers. We strongly encourage code deposition in a community repository (e.g. GitHub). See the Nature Portfolio guidelines for submitting code & software for further information.

Data

Policy information about availability of data

All manuscripts must include a data availability statement. This statement should provide the following information, where applicable:

- Accession codes, unique identifiers, or web links for publicly available datasets
- A description of any restrictions on data availability
- For clinical datasets or third party data, please ensure that the statement adheres to our policy

We utilised 27,136 structures from the MP-20 training dataset as the target set for embedding extraction. While MACE models provide a native function, other architectures required custom interfaces to access the latent layers. An extraction script (https://github.com/WMD-group/PlatonicRep) was used to generate a total of 282,847 atomic embeddings per model. All extracted model-wise embeddings have been archived to facilitate anchor set generation and reproducibility in Zenodo DOI: 10.5281/zenodo.17721681.

Research involving human participants, their data, or biological material

Policy information about studies with human participants or human data. See also policy information about sex, gender (identity/presentation), and sexual orientation and race, ethnicity and racism.

Reporting on sex and gender	This study doesn't involve human sex and gender.
Reporting on race, ethnicity, or other socially relevant groupings	This study doesn't involve human race, ethnicity, or other socially relevant groupings.
Population characteristics	This study doesn't involve human population characteristics.
Recruitment	This study doesn't involve recruitment.
Ethics oversight	This study doesn't involve human ethics.

Note that full information on the approval of the study protocol must also be provided in the manuscript.

Field-specific reporting

Please select the one below that is the best fit for your research. If you are not sure, read the appropriate sections before making your selection.

☐ Life sciences ☐ Behavioural & social sciences ☒ Ecological, evolutionary & environmental sciences

For a reference copy of the document with all sections, see nature.com/documents/nr-reporting-summary-flat.pdf

Ecological, evolutionary & environmental sciences study design

All studies must disclose on these points even when the disclosure is negative.

Study description	Computational comparative analysis of latent representations across seven independently-trained machine learning interatomic potentials. Design: systematic variation of anchor set size (K = 3, 8, 20, 50, 100, 200, 400) and sampling strategy (DIRECT vs. random). Experimental units: 282,847 atomic embeddings extracted from 27,136 crystal structures (MP-20 dataset) across seven models. No biological replicates; computational reproducibility ensured via fixed random seeds {0, 42, 12345}.
Research sample	Research sample consists of atomic-level latent representations (embeddings) extracted from seven foundation machine learning models applied to 27,136 inorganic crystal structures from the Materials Project MP-20 dataset. Sample choice rationale: MP-20 provides broad coverage of inorganic chemical space and is a standard benchmark dataset; the seven models represent diverse architectural approaches (equivariant vs. non-equivariant, conservative vs. non-conservative) trained on overlapping but distinct datasets (MPtrj, OMat24), enabling assessment of representational convergence across model families.
Sampling strategy	Sample size (27,136 structures, 282,847 atoms) was determined by the complete MP-20 dataset, chosen to ensure comprehensive coverage of the inorganic chemical space represented in Materials Project data. Anchor sets were sampled using DIRECT stratified clustering to maximize chemical diversity. Anchor set size convergence was empirically validated by testing K = 3 to 400; representations stabilized at K = 100 (Figs. 3, S2-S3), confirming sufficiency.
Data collection	Atomic embeddings were computationally extracted from the latent layers of seven pre-trained foundation models using custom Python interfaces. For MACE models, the native get_descriptor() function was used; for other architectures (SevenNet, Orb-v3), custom extraction scripts accessed intermediate layer outputs. All extractions were performed programmatically with fixed random seeds for reproducibility. Extracted embeddings were archived in Zenodo (DOI: 10.5281/zenodo.17721681).
Timing and spatial scale	This is a cross-sectional computational study with no temporal sampling component. All embeddings were extracted from pre-trained model checkpoints in a single computational session (2025). Spatial scale: atomic-level representations in high-dimensional

	latent space (128-256 dimensions depending on architecture), projected into unified K-dimensional anchor space. Physical structures span the inorganic materials space represented in the Materials Project.
Data exclusions	No data were excluded from the analyses. All 282,847 atomic embeddings extracted from the 27,136 MP-20 structures were included in the unified representation analysis. The complete dataset ensures unbiased assessment of representational alignment across the full chemical space covered by the foundation models.
Reproducibility	Computational reproducibility was ensured through: (1) fixed random seeds {0, 42, 12345} for anchor sampling (Table S1); (2) version-controlled code deposited in public GitHub repository (https://github.com/WMD-group/PlatonicRep); (3) archived embeddings in Zenodo (DOI: 10.5281/zenodo.17721681); (4) documented model versions and hyperparameters. Anchor sampling was repeated across three random seeds, confirming convergence of DIRECT strategy. All computational experiments were successfully reproducible within numerical precision (tolerance 1e-6 for floating-point operations).
Randomization	Randomization is not applicable to this computational study. All seven foundation models were analyzed using the complete MP-20 dataset (27,136 structures) without group allocation. Anchor selection employed stratified DIRECT sampling to maximize chemical diversity rather than random allocation. Model-to-model comparisons were controlled by ensuring identical input structures across all models, eliminating structural covariates. Random seeds {0, 42, 12345} were used solely to assess sampling variance in anchor selection, not for experimental group allocation.
Blinding	Blinding is not relevant to this computational study. All analyses involved deterministic extraction and transformation of embeddings from pre-trained models with fixed parameters. There were no subjective assessments, human annotations, or treatment conditions that could introduce observer bias. Model identities were known throughout analysis as cross-model comparison was the explicit objective of the study. Computational reproducibility (fixed random seeds, version-controlled code) replaces the role of blinding in controlling for bias.

Did the study involve field work? ☐ Yes ☒ No

Reporting for specific materials, systems and methods

We require information from authors about some types of materials, experimental systems and methods used in many studies. Here, indicate whether each material, system or method listed is relevant to your study. If you are not sure if a list item applies to your research, read the appropriate section before selecting a response.

Materials & experimental systems

n/a	Involved in the study
☒	☐ Antibodies
☒	☐ Eukaryotic cell lines
☒	☐ Palaeontology and archaeology
☒	☐ Animals and other organisms
☒	☐ Clinical data
☒	☐ Dual use research of concern
☒	☐ Plants

Methods

n/a	Involved in the study
☒	☐ ChIP-seq
☒	☐ Flow cytometry
☒	☐ MRI-based neuroimaging

Plants

Seed stocks	This study does not involve plants, seed stocks, or biological specimens. The research analyzes computational representations (embeddings) of inorganic crystal structures from the Materials Project MP-20 dataset, which contains synthesized inorganic materials such as metal oxides, semiconductors, and ceramics. No plant material was used or collected.
Novel plant genotypes	This study does not involve plants or plant genotypes. No novel biological organisms were generated. The research focuses on machine learning model representations of inorganic crystalline materials.
Authentication	This study does not involve plants, seed stocks, or biological authentication. Model authenticity was verified through: (1) use of official pre-trained model checkpoints from published sources (MACE-MP-0, SevenNet, Orb-v3); (2) version documentation for all models; (3) validation that models reproduced expected performance on benchmark datasets as reported in original publications. The dummy control model (random weights, no training) served to verify that observed patterns arise from learned representations rather than architectural artifacts.
